# Effects of the salinity-temperature interaction on seed germination and early seedling development: a comparative study of crop and weed species

**DOI:** 10.1186/s12870-023-04465-8

**Published:** 2023-09-22

**Authors:** Nebojša Nikolić, Aurora Ghirardelli, Michela Schiavon, Roberta Masin

**Affiliations:** 1 Department of Agronomy, Food, Natural Resources, Animals and Environment - DAFNAE, University of Padua, Legnaro (PD), 35020 Italy; 2 Department of Agricultural, Forest and Food Sciences - DISAFA, University of Turin, Grugliasco, TO 10095 Italy

**Keywords:** Salinity, Weeds, Crops, Germination, Early development, Climate change

## Abstract

**Background:**

Weeds represent a great constraint for agricultural production due to their remarkable adaptability and their ability to compete with crops. Climate change exacerbates the abiotic stresses that plants encounter. Therefore, studying plant responses to adverse conditions is extremely important. Here, the response to saline stress at different temperatures of three weed species (*Chenopodium album*, *Echinochloa crus-galli* and *Portulaca oleracea*) and three crops (maize, soybean and rice) was investigated.

**Results:**

The germination percentage of soybean notably decreased as salinity and low temperatures increased. In contrast, maize and rice consistently maintained a high germination percentage, particularly when subjected to low salinity levels. Regarding weed species, the germination percentage of *C. album* was not significantly affected by salinity, but it decreased in *E. crus-galli* and *P. oleracea* with increasing salinity. The mean germination time for all species increased with salinity, especially at lower temperatures. This effect was most pronounced for soybean and *E. crus-galli*. *C. album* exhibited significant reduction in stem growth with high salinity and high temperatures, while in *E. crus-galli* stem growth was less reduced under similar conditions.

**Conclusion:**

This study showed that successful germination under saline stress did not ensure successful early development and emphasizes the species-specific nature of the temperature-salinity interaction, perhaps influenced by intraspecific variability. Increasing salinity levels negatively impacted germination and seedling growth in most species, yet higher temperatures partially alleviated these effects.

**Supplementary Information:**

The online version contains supplementary material available at 10.1186/s12870-023-04465-8.

## Background

Salt-affected soils (saline and sodic) are defined as the soils containing high concentrations of soluble salts, which adversely affect the growth and productivity of most crops [1, 2]. The detrimental effects of salinity can be accentuated with increasing temperature [3]. These soils hold an electrical conductivity (EC) above 4 dS/m (deciSiemens per meter), but the threshold should be lowered to 2 dS/m, due to the negative outcomes this level of EC has on different plant species [4–6]. Salt-affected soils represent approximately 20 to 30% of total arable lands [7, 8]. This percentage is expected to increase in the future due to low precipitation, high surface evaporation, weathering of native rocks, irrigation with saline water, and poor agricultural practices [8, 9]. The conditions leading to soil salinization typically occur in arid and semiarid areas of South and Southeast Asia [1, 10], and in various Mediterranean regions [11]. Also, the rise in mean sea level due to global climate change can lead to a secondary salinization phenomenon, resulting from irrigating crops with saline water [10, 12, 13]. Secondary salinization is predicted to affect 30% of arable land by the end of the next decade, and more than 50% by the end of the century [14]. Therefore, it is a matter of concern in agriculture, as it can impair crop production on a global scale and negatively impact the nutritional needs of a growing world population [15].

Currently, salinity management in agroecosystems is concerned both with mitigating the sources of salt stress that impact crops and with enhancing the salt tolerance mechanisms of plants [16]. Salt stress has the potential to affect plants at multiple levels: cellular, biochemical and physiological. It can have detrimental effects on germination, growth, and reproduction. In particular, salt stress poses a significant threat to photosynthesis in plants, which can be attributed to various mechanisms. These include changes in enzymatic activities, suppression of chlorophyll biosynthesis, impairment of the photosynthetic apparatus, diminished electron flow from photosystem II (PSII) to photosystem I (PSI), dissipation of heat energy through non-photochemical processes, and reduced CO_2_ influx due to stomatal closure [17]. In some plants, exposure to salinity can also give rise to genetic variations [18].

Salt-induced damage in plants is primarily caused by hyperosmotic stress and ion imbalance. This occurs due to excessive accumulation of sodium (Na^+^) and chloride (Cl^−^) ions, coupled with a concomitant reduction in potassium (K^+^) and calcium (Ca^2+^). As a consequence, oxidative stress and changes in protein conformation are induced [1, 19].

Salt-tolerant plants activate osmotic adjustments through increased production of osmolytes (e.g., proline, sugar alcohols). In addition, they can efficiently regulate ion membrane transport so as to maintain cellular osmotic and turgor pressure. In particular, they enhance the uptake of K^+^, while decreasing the uptake of Na^+^ and its transfer to the leaves. Osmoprotective and ion-detoxification strategies also consist in the removal of Na^+^ from the cytosol and its compartmentation in the vacuoles, and in higher K^+^ retention to maintain optimal K^+^/Na^+^ ratio [20].

The regulation of ion channels and transporters for Na^+^ and K^+^ is influenced by the microtubules of the cytoskeleton, which plays a crucial role in the uptake and distribution of these ions in plants [21]. Furthermore, the cytoskeleton contributes to maintaining cell shape and integrity under salt stress conditions and interacts with calcium signaling pathways, thereby modulating signal transduction and calcium-mediated stress responses. Disruption of cytoskeleton organization due to salinity can lead to the accumulation of reactive oxygen species (ROS) within plant cells, which accumulation serves as an initial signal triggering salt stress responses in plants [21]. Indeed, ROS serve as signalling molecules at low concentration. However, when their levels become high, they have the potential to harm cellular components through oxidative damage. In response, plants activate antioxidant defense mechanisms involving enzymes such as superoxide dismutase (SOD), catalase (CAT), and peroxidases, which play a crucial role in scavenging and detoxifying ROS, mitigating their harmful effects [22].

Salinity stress elicits a diverse array of signaling pathways within plant cells, enabling them to sense and effectively respond to the stress [23]. Notably, the mitogen-activated protein kinase (MAPK) cascades and the phytohormones abscisic acid (ABA) and ethylene play crucial roles in transmitting the stress signals and regulating downstream stress-responsive genes. MAPKs and the pathways involving abscisic acid (ABA) and ethylene regulate plant growth, stomatal closure, osmotic adjustment, ion balance, and ROS scavenging by orchestrating the activation of specific stress-responsive genes and proteins [21, 24, 25]. Several genes, including NHX and AVP, play a role in facilitating the sequestration of Na^+^ into leaf vacuoles, while HKT and SOS1 aid in preventing the long-distance transport of Na^+^. Genes such as P5CS, OTS, MT1D, M6PR, S6PDH, and IMT1 regulate the synthesis of compatible solutes in the cytoplasm. Additionally, genes like SOS3 and SnRKs participate in modifying long-distance signaling processes. Furthermore, genes such as ERA1, PP2C, AAPK, and PKS3 contribute to the maintenance of efficient photosynthesis by minimizing stomatal closure [23]. Abscisic acid also exhibits cross-talk with the salicylic acid signaling pathway and this interplay may coordinate plant responses to salt stress, modulating salt-responsive genes and physiological processes [21, 26]. Salicylic acid complements these effects by mitigating the adverse consequences of salt stress. It promotes photosynthesis and enhances the activity of the cellular antioxidant system, offering protection against oxidative damage caused by salt stress [26, 27]. It also prevents the decline of auxin and cytokinin levels in salt-stressed plants, enabling proper root development and enhancing crop productivity [26].

The effect of salt stress on seed germination and early plant development can vary between crops and between varieties within the same species [13, 28]. Resistance to salinity is therefore a critical trait for natural selection [29–31]. The research present in the literature is mostly focused on the combined effect of temperature and salinity on the growth and development of crop species [32, 33]. Indeed, temperature is major factor influencing seed germination and plant development [34], and different species have an optimal temperature under which seeds germinate best [3]. Under low temperature, plant growth can be inhibited due to increased ROS accumulation, alteration in primary metabolism, and reduced efficiency of processes and enzymatic reactions crucial for growth and development [35]. In response to low temperature, plants undergo adjustments in cell membrane lipid composition [36] and experience an elevation in cytosolic Ca^2+^ levels. Calcium-dependent protein kinases (CPKs) sense the fluctuations in cytosolic Ca^2+^ levels and interact with various downstream signaling molecule like hormones, mitogen-activated protein kinases (MPKs), and ROS. This signaling cascade plays a significant role in plant acclimation to cold stress [37]. In recent studies, several co-expressed genes were identified in quinoa (*Chenopodium quinoa* Willd) under different treatments, including NaCl and low temperature [38]. This finding suggests an overlap in the plant’s responses to these conditions, indicating the activation of common transcriptional signatures.

The general trend reported in the literature seems to indicate that crops can tolerate salinity up to a certain temperature after which germination starts declining, and seedling establishment and growth are altered [32, 33, 39]. As for the weed species, the effects of salinity and temperature are often assayed separately. However, given the role of temperature in seed germination and seedling establishment, more studies should focus on the combined effects of salinity and temperature on these processes in weeds.

Weed species, in particular, are usually more tolerant to abiotic stresses than crops, but it is not possible to formulate general assumptions on their responses to salinity in combination with temperature [40, 41]. Thus, the responses of each individual weed species must be assayed. Furthermore, the comparison of crop and weed responses during germination with the same combinations of salinity and temperature is worth studying to improve knowledge of crop-weed interactions under a changing climate scenario.

Compared to their wild ancestors, modern crops appear to be more sensitive to salinity, probably due to a trade-off during the selection process in which the salinity tolerance trait was discarded in favor of higher productivity. Adverse effects of salinity have been described in major staple crops, such as maize (*Zea mays* L.), soybean (*Glycine max* L.), and rice (*Oryza sativa* L.). Only a few crops have been reported to be more salt-tolerant than weeds, such as sorghum compared with *Striga hermonthica* [42].

Most studies indicate that weed species are more resilient than crops to salinity likely because of greater intraspecific genetic variability and more adaptive strategies developed during their evolution [16]. For example, *Chenopodium album* produces both black and brown seeds. Black seeds will preferentially be generated under salt stress due to their lower dormancy and higher tolerance to salinity than brown seeds [43]. Furthermore, Watkinson et al. [44], suggested that the highly salt-tolerant weeds *C. album*, *Echinochloa crus-galli* and *Portulaca oleracea* could become more widespread in the Mediterranean area with increasing soil salinity levels, and possibly also more invasive. This is because the three weed species exhibit high adaptive potential and plasticity [34, 45, 46]. *C. album* and *P.oleracea* can display high resilience to germination in saline conditions [46, 47]. *P.oleracea*, in particular, is defined as a halophyte [48]. On the other hand, *E. crus-galli *shows very different responses to salinity, probably due to its high intraspecific variability [49–51]. Different studies report that *P. oleracea*, *C. album* and *E. crus-galli* are already widespread weeds in different parts of the world. In Italy and the rest of the Mediterranean area, they largely occur especially in summer crops such as maize and soybean, where they show high competitiveness with the crops. In addition, E. *crus-galli* is one of the main troublesome weeds in paddy soils, as it grows more vigorously than rice plants and competes better for nutrient resources.

The competition between crops and weeds could be very unbalanced in saline environments, starting from the seed germination stage [52–55]. Furthermore, if weeds are more resilient than crops, as the data from the literature indicate, the competition between them could be exacerbated by suboptimal temperatures. To test this hypothesis, we conducted germination assays and growth tests of three weed species (*C. album*, *P. oleracea*, and *E. crus-galli*) and three staple crops (maize, soybean, and rice) in a saline environment using five different salinity levels in combination with three different temperatures.

## Results

The ANOVA revealed a significant effect of all factors and their interactions on seed germination (Table 1).

**Table 1:** Effects of species, salinity, temperature, and their interactions on germination

Factors	p-value
Species	0.0000
Salinity	0.0000
Temperature	0.0000
Species*Salinity	0.0000
Species*Temperature	0.0000
Salinity*Temperature	0.0016
Spec*Sal*Temp	0.0000

Spec-species, Sal-salinity, Temp-temperature

**Fig. 1:**
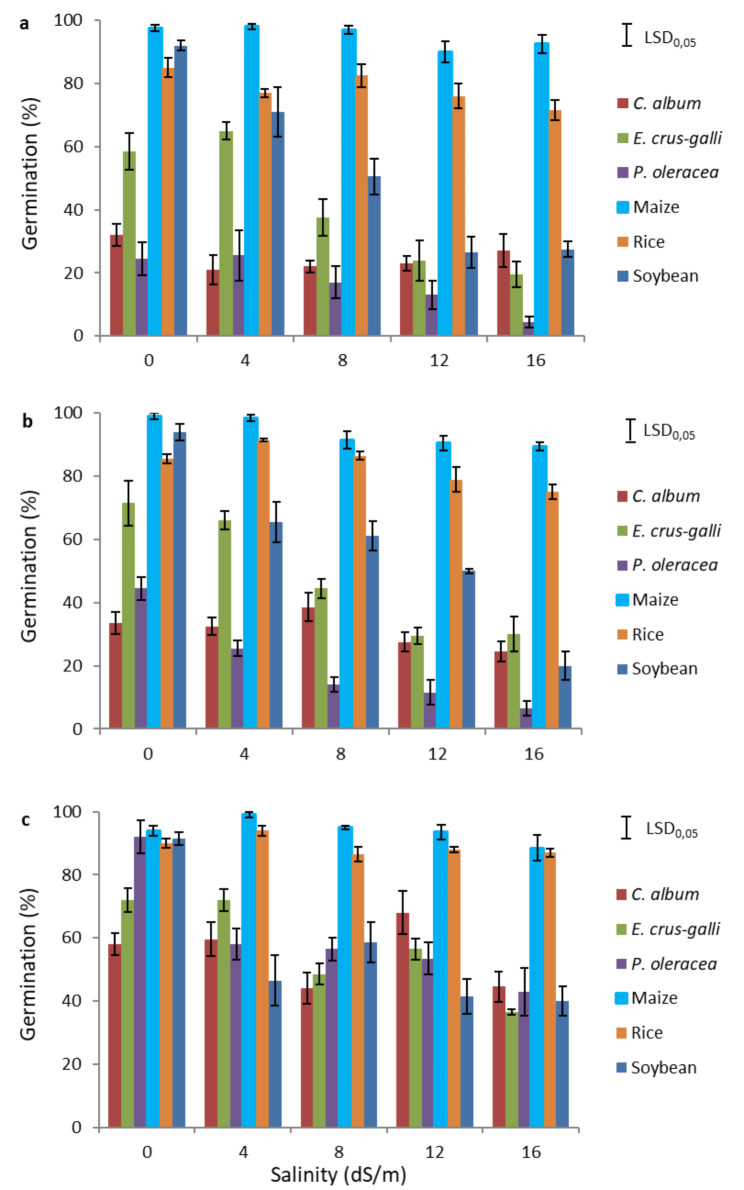
Germination percentages of the three weed species *Chenopodium album*, *Echinochloa crus-galli*, and *Portulaca oleracea*, and the three crop species Maize (*Zea mays*), Rice (*Oryza sativa*), and Soybean (*Glycine max*) at different salinity levels and temperatures of 12 °C **(a)**, 15 °C **(b)** and 18 °C **(c)**

In soybean, *E. crus-galli* and *P. oleracea*, the germination percentage decreased with increasing salinity at each temperature (Fig. 1).

With respect to the effect of the salinity-temperature interaction on seed germination, the germination percentage increased with increasing temperatures at almost every salinity level. An exception was for seeds germination with the highest salinity level (16 dS/m), at which an increase in germination was observed at 18 °C, but not at 12 and 15 °C (Additional Figure S1).

Crop species had a relatively even and high germination percentage (Additional Table S1), with maize and rice performing best at every temperature. However, maize seeds exhibited the highest germination percentage with significant reduction only above 12 dS/m, while low salinity levels appeared to increase germination. Soybean was the most sensitive crop to salinity, showing a 21–45% decline in germination even at the lowest salinity level (4 dS/m) depending on the temperature. When soybean seeds were sown in the low-saline medium (4 dS/m), the negative effect of increasing temperature on germination was evident, with reductions of 28% at 15 °C and 45% at 18 °C.

In contrast to the pattern observed for crops, the germination percentages of weed species were highly variable as a function of salinity and temperature (Additional Table S2). At low temperatures in the absence of NaCl, *E. crus-galli* had a higher germination percentage than the other two weeds. However, differences in germination between the weed species were less pronounced with increasing temperature. *E. crus-galli* seeds germinated well with low salinity, less so at salinity levels of 8 dS/m or higher. A similar trend was observed for *P. oleracea*, with seed germination inhibited not only by salinity but also by low temperatures. Specifically, there was no variation in germination percentage between the seeds in the low-salinity medium (4 dS/m) and the seeds in the control medium at 12 °C. However, variations in germination did occur at higher temperatures (15 and 18 °C). The germination percentage of *C. album* was generally low (maximum 68% at 18 °C at 12 dS/m salinity level), this species showed little or no reduction with increasing salinity, indicating that the seeds of this species were less sensitive to salinity during the germination process.

If we look at salinity alone, its effect on the germination of each species regardless of temperatures is even more evident (Additional Figure S2).

Weed and crop species showed different levels of salinity tolerance, even though their germination percentages were different even without salt stress. The most salt-tolerant species were maize among the crops, and *C. album* among the weeds, while soybean and *E. crus-galli* were the most sensitive.

The results obtained from the ANOVA performed on the mean germination time (MGT) of the six species showed a significant effect of all factors and their interactions (Table 2).

**Table 2:** Effects of species, salinity, and temperature, and their interactions on mean germination time (MGT).

Factors	p-value
Species	0.000
Salinity	0.000
Temperature	0.000
Species*Salinity	0.032
Species*Temperature	0.000
Salinity*Temperature	0.002
Spec*Sal*Temp	0.003

Spec-species, Sal-salinity, Temp-temperature

**Fig. 2:**
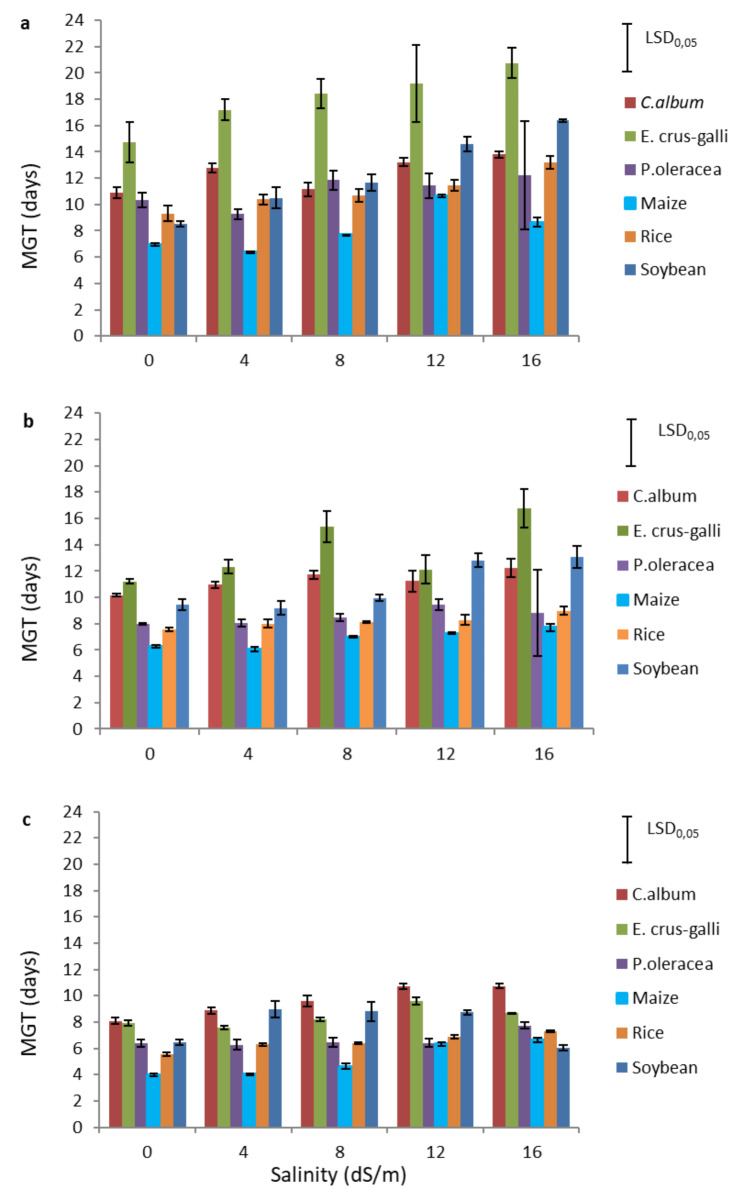
Mean germination time (MGT) of the three weed species *Chenopodium album*, *Echinochloa crus-galli*, and *Portulaca oleracea*, and the three crop species Maize (*Zea mays*), Rice (*Oryze sativa*), and Soybean (*Glycine max*) at different salinity levels and different temperatures: 12 °C **(a)**, 15 °C **(b)**, and 18 °C **(c)**

The MGT of all species generally increased with increasing salinity, and this effect was more pronounced at the lower temperatures (12 and 15 °C), particularly in soybean and *E. crus-galli* (Fig. 2). In the control conditions (0 dS/m), the MGT was lower for crops than for weeds. Among the crops, maize and rice had a lower MGT than soybean, and were less affected by increased salinity in terms of delayed germination, especially at lower temperatures. For example, at 12 °C, the MGT for soybean at 0 dS/m was 9 days, but increased to 16 days at 16 dS/m. On the other hand, at the same temperature the MGT for rice was 9 days at 0 dS/m, rising to 13 days at 16 ds/m, and for maize it was 10 days at 0 dS/m rising to 12 days at 16 dS/m, maize results, however, showed high variability, as can be seen in Additional Table S3. Among the weeds, *P. oleracea* seeds germinated the fastest at every temperature and salinity level, with an MGT ranging from 7 days at 12 °C to 4 days at 18 °C (values at 0 dS/m). *E. crus-galli* seeds were in general the slowest to germinate, with an MGT ranging from 15 days at 12 °C to 8 days at 18 °C (values at 0 dS/m), and was also the most sensitive weed species to increased salinity, especially at low temperature, with an MGT at 12 °C of 15 days at 0 ds/m, and 21 days at 16 dS/m, data shown in Additional Table S4.

Overall, our results show that MGT decreased with increasing temperature, regardless of the salinity level applied.

**Fig. 3:**
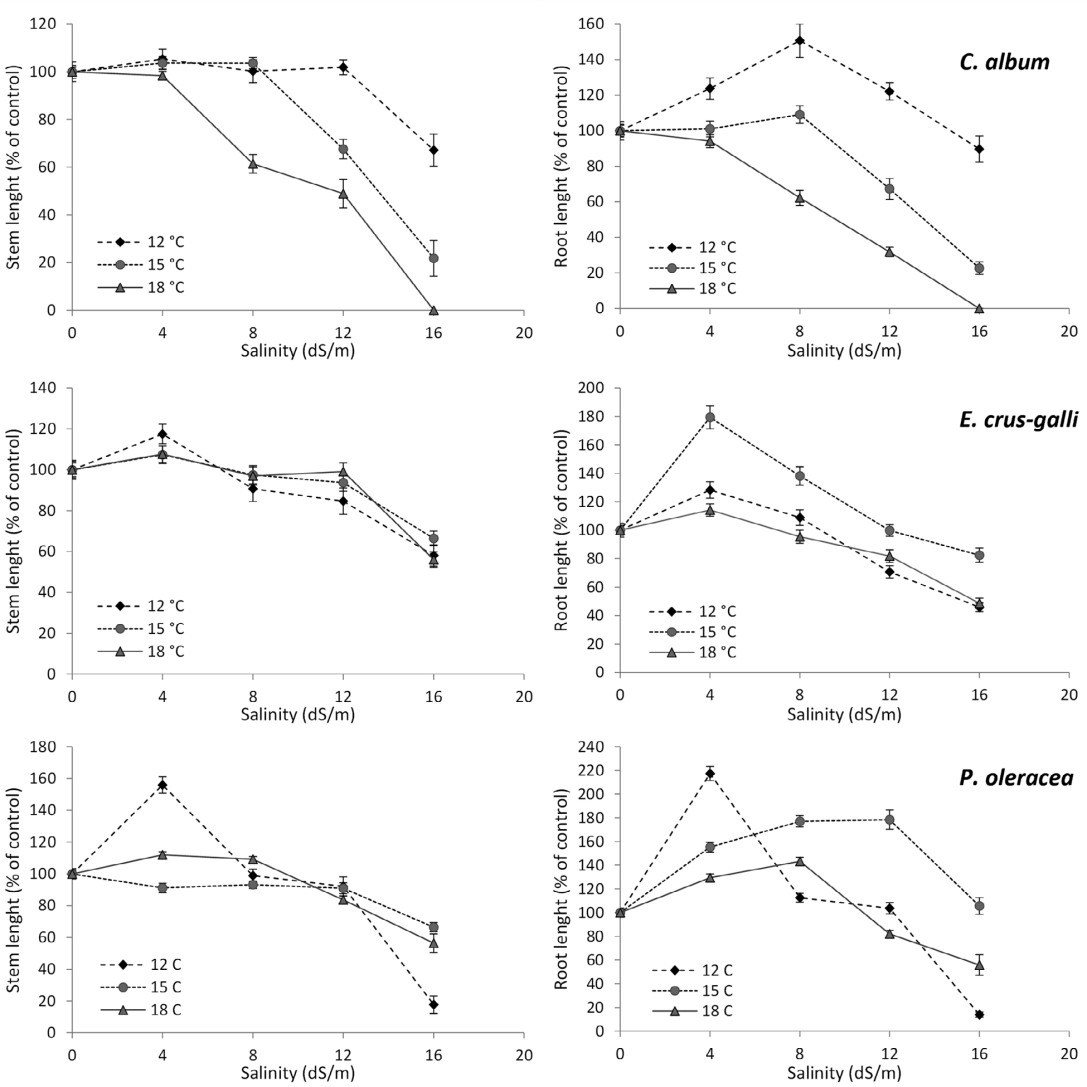
Stem and root length (in percentage of the control), of *Chenopodium album*, *Echinochloa crus-galli* and *Portulaca oleracea* at different salinity levels and temperatures (root and stem control length can be seen in Additional table S5)

**Fig. 4:**
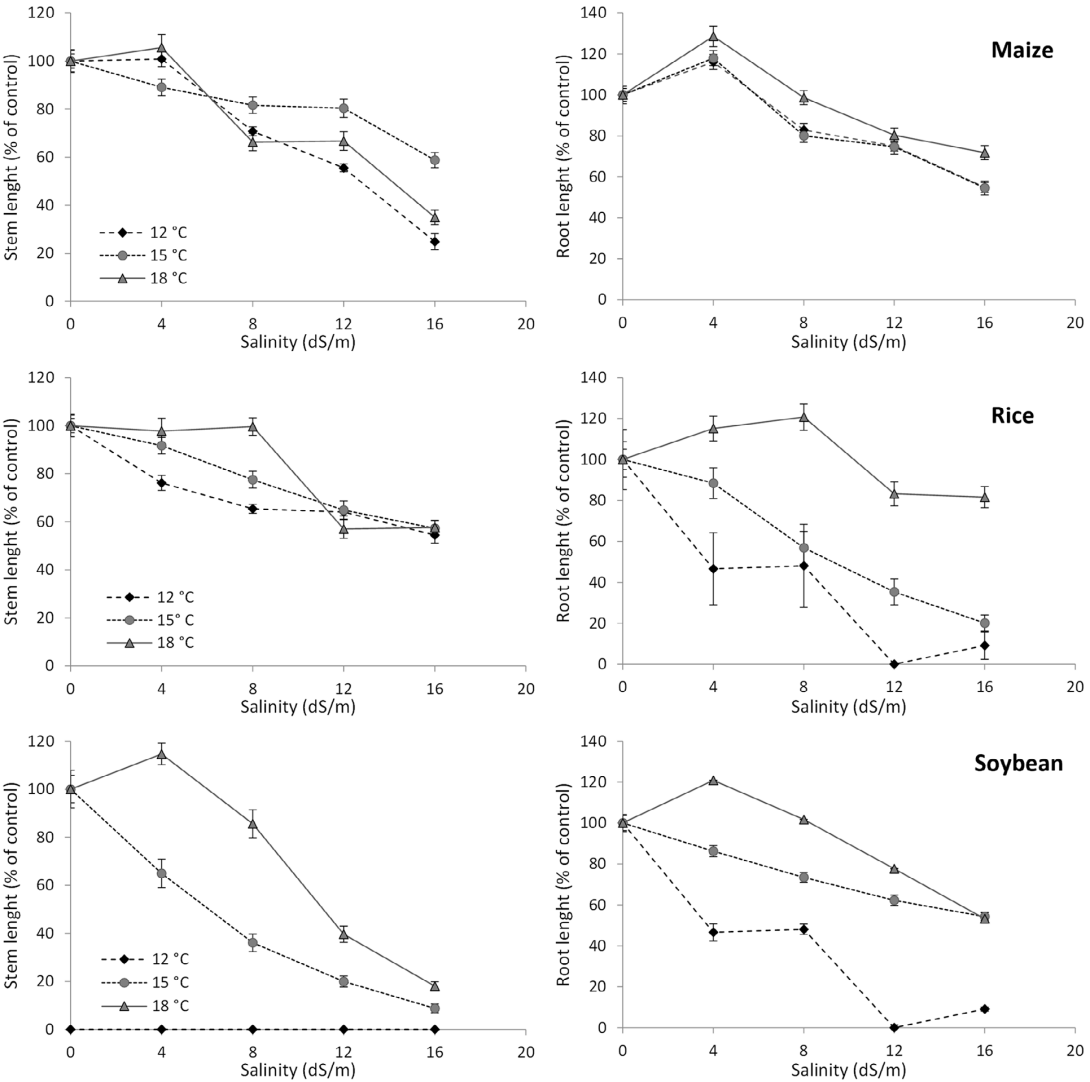
Stem and root length (in percentage of the control), of maize (*Zea mays* L.), rice (*Oryza sativa* L.) and soybean (*Glycine max*) at different salinity levels and temperatures (root and stem control length can be seen in Additional table S5)

Growth test results indicated that the six species assayed in this study were able to develop at different salinity and temperature levels. However, successful germination does not always imply successful early-stage development (Figs. 3 and 4). For example, *C. album* was the least affected by salinity in terms of germination, but its stem growth was reduced with increased salinity (Fig. 3), especially with a combination of high salinity (16 dS/m) and high temperature (18 °C), where no stem elongation was observed. Root elongation followed a similar trend, except that it improved at the low levels of salinity, especially at 12 °C. The stem growth of *E. crus-galli*, the weed most sensitive to salt stress in terms of germination, decreased with increased salinity, but not with temperature. The root elongation of this species followed a trend similar to stem growth, except that at low salinity levels and 15 °C root development was better than at 0 dS/m. The stem growth of *P. oleracea*, on the other hand, was stimulated by low temperature (12 °C), at low salinity (4 dS/m), while only slightly altered by 15 and 18 °C. Increasing the salinity level determined the inhibition of stem growth, which was sharper at low temperature (Fig. 4). Root elongation was stimulated by all temperatures under low salinity. At 15 and 18 °C, the increase of root elongation was observed under salinity levels up to 12 dS/m and 8 dS/m, respectively.

Regarding the crops, the stem length of maize seedlings was reduced by medium and high salinity, particularly at 12 and 18 °C (Fig. 4). Root elongation showed the same trend, although it was less affected by salt stress than stem growth, and was less reduced at high temperatures. Stem elongation of rice seedlings decreased with rising salinity, but was barely affected by temperature. Like maize, the reduction in stem growth was not very pronounced, especially when compared to some of the weed species. Nevertheless, root elongation was more affected by temperature, especially at 12 and 15 °C, even at low salinity levels. The stem and root growth of soybean was consistent with germination, confirming this species as the least tolerant of the crops tested in this study to salinity (Fig. 4). The growth of the soybean stem was highly dependent on temperature: it was completely inhibited at the lowest temperature (12 °C), and significantly reduced at higher temperature with increasing salinity. In contrast, the roots of soybean were able to develop at 12 °C and were less inhibited by salinity.

## Discussion

This study shows that the effect of salt stress on seed germination and early seedling development depends on the species, the degree of salinity, the temperature, and the combination of salinity with temperature. Soybean was found to be the least tolerant of the crops to salinity. This is consistent with literature classifying soybean as a moderately salt-sensitive species with its yield reduced by soil salinity above 5 dS/m [56]. Our results concerning germination are in agreement with those of [57], who reported a significant reduction in germination rates for soybean seeds exposed to salt levels of 4.5 dS/m or higher. Such effect may be attributed to NaCl capacity to stimulate the biosynthesis of ABA while concurrently inhibiting gibberellic acid (GA) in this species, as previously reported [58]. In addition, salt stress could hamper the seed’s ability to take up water by lowering the osmotic potential of the external medium, thus preventing its hydration and germination [59]. It can also lead to sodium and/or chloride toxicity in the embryo or disrupt protein synthesis, further exacerbating the negative effects on seed germination and development [60]. However, we observed that high salinity had a greater effect on the MGT of this species, contrary to what was reported by [61], who found no significant increase in MGT with NaCl concentrations below 16 dS/m. This discrepancy may be due to the fact that germination was tested at different temperatures in the two studies: [61] used an experimental temperature close to the optimum, whereas the temperatures we applied were closer to the base temperature. Therefore, it is possible that suboptimal temperature in our study might have accentuated the severity of salt stress in soybean, which typically germinates well at temperature above 10 ^o^C. Coherent with this hypothesis, the effect of salt stress on MGT was much less severe at 18 °C than at lower temperatures.

Unlike soybean, maize appeared to be relatively tolerant to salinity at the germination stage, despite being classified as a moderately sensitive crop in the literature [60]. Indeed, salt stress is known to affect maize development at different stages, with germination and stand establishment being particularly vulnerable compared to later developmental stages [60]. In addition, maize growth parameters are often significantly reduced as a function of increasing salinity [60, 62, 63]. The high tolerance of maize seeds to salinity in our study could be due to the particular cultivar used, its sensitivity to salt being unknown at the time of the experiment. Maize is highly polymorphic and is considered to have the highest genetic variability among crops [64, 65], with its various cultivars differing in salt tolerance at the germination stage [63, 66]. The fact that seed germination was not delayed with increased salinity supports the hypothesis that our cultivar was probably salt-tolerant at this stage. In this case, it is expected that germinating maize seedlings activated various tolerance strategies to mitigate salt stress, like exclusion of excessive Na^+^ or its compartmentation into vacuoles, or the upregulation of defense genes and β-expansin proteins to sustain growth [60].

Rice was found to be relatively tolerant to salinity. The capacity to tolerate salt by rice is generally achieved through two principal mechanisms, i.e. ion exclusion preventing the excess accumulation of Na^+^ and Cl^−^ in leaves, and osmotic tolerance via sequestration of Na^+^ in the vacuole, synthesis of osmolytes and production of antioxidant enzymes [23, 67]. Our results are consistent with those reported by other authors who observed rice tolerance to salt at concentrations up to 10 dS/m [68] or 16 dS/m [69, 70] at the germination phase, with no substantial reduction in the germination percentage and speed. As in the case of maize, many authors have reported varying degrees of salt tolerance in different rice varieties, with some of the most common cultivars being extremely salt-sensitive [13, 71–73]. In the case of salt-sensitive rice varieties, transplanting aged seedlings could be a possible option to alleviate the salinity at the seedling stage and reduce competition with salt-tolerant weeds.

Among weeds, *C. album* has been definitely confirmed as a salt-tolerant species [74], especially with respect to seed germination [46]. Our findings are consistent with those of [75], who found *C. album* to be salt tolerant up to 20 dS/m at the germination stage, with no steep increase in MGT and a germination percentage of 40% even at a salinity of 30 dS/m. However, high salinity levels exerted a negative effect on plant growth in the early stages of plant development, especially at lower temperature. Interestingly, different results can be obtained when salinity is applied once the seedling stage has been established. In a recent study by [74], *C. album* was reported to be highly tolerant to 150 mM NaCl (above 10 dS/m), when the salt was applied to 10 cm high-plants. In this case, the weed resilience to salt was mainly due to elevated initial K^+^ concentration and abundant K^+^ delivery to the shoot, high accumulation of phenolics and proline, high antioxidant activity and low lipid peroxidation in the weed. One possible hypothesis is that at a later stage of development the weed might be more efficient in activating the mechanisms involved in salinity stress tolerance.

*Portulaca oleracea* is considered to be either a halophyte or a moderately tolerant species, depending on the scientific source [76, 77]. This is because the wide range of genetic diversity within *P. oleracea* populations allows for the presence of individuals that vary in tolerance to salinity stress. Furthermore, the capacity of *P. oleracea*, a C4 plant, to engage in Crassulacean Acid Metabolism (CAM), enhances the plant water-use efficiency, further contributing to its resilience and ability to thrive in saline environments [78]. In our study, however, we found a significant reduction in the germination percentage of this species, which could be due to multiple factors, including intraspecific variation, the experimental temperatures being lower than the optimum, and/or to the specific traits of the ecotype used. The considerable morphological and physiological plasticity exhibited by *P. oleracea* [79] may have additionally contributed to the observed variations in germination. Therefore, its capacity to tolerate high-salt concentrations cannot be generalized to the species, rather to the ecotype.

Our results indicate *E. crus-galli* as the most sensitive weed to salinity, which is in contrast to the findings of [80] and [50], who classified this species as salt tolerant. It must be noted that in the study by [50] *E. crus-galli* was not subjected to NaCl since the seed stage, but 10 days after sowing, i.e. when 50% of the seedlings reached 5 cm height. Thus, it is possible that the weed at this stage was more efficient in activating salt-tolerance mechanisms, and thus less salt-sensitive. In addition, [49] found substantial variation in the response to salinity among different ecotypes of *E. crus-galli* from Italy. However, it was noted that germination remained largely unaffected for all ecotypes up to 250 mM NaCl (above 20 dS/m). The ecotype we used was able to tolerate salt stress up to 4 dS/m, but seed germination was significantly affected at and above 8 dS/m. This could be due to the experimental temperatures used in our study, perhaps all suboptimal for the weed, as suggested by the decreasing gap between salinity levels with increasing temperature.

The effects of salinity and temperature at the early growth stage were on the whole consistent with the effects on seed germination, with a few exceptions. Those species that appeared to be salt tolerant at the germination stage (i.e. maize, rice, and *C. album*) showed significantly reduced root and stem lengths with increasing salinity. This discrepancy can be explained by the fact that many plant species exhibit different responses to salt stress depending on the growth stage, among which are the activation of salt-stress responsive genes, the induction of Na^+^ and Cl^−^ efflux root transporters, the accumulation of osmolytes and Na^+^ vacuolar sequestration [23, 67, 81].

Rice and maize are considered to be more vulnerable to salinity at the early growth stages [13, 64] than at the germination stage [63, 82]. This means that at the seedling stage, salt stress significantly impacts sensitive metabolic processes, such as photosynthesis (e.g., via reduced chlorophyll synthesis, gas exchanges and electron transport rate), in these crops. In the case of *C. album*, temperature also played a key role: at higher temperatures a greater percentage of seeds germinated in all treatments, but salt stress inhibited further growth, resulting in a large number of slowly developing seedlings. Soybean at the seedling stage was even more sensitive to salinity than at the germination stage, confirming the findings of [61], who reported that the early growth stages of this species are affected by lower salinity levels. Like soybean, *P. oleracea* and *E. crus-galli* were rather sensitive to salt at the seedling stage, in agreement with [83], [80], and [84]. Despite the general trend of root length reduction, root growth in all the weed species was slightly stimulated at low salinity levels (4–8 dS/m). Such a phenomenon is known as the hormetic effect [85], previously observed in numerous weed species, including *P. oleracea* and *E. crus-galli* [48, 84].

Although many sources in the literature report salt-tolerance traits in maize and rice, these crops are generally more sensitive to salinity than most of the weeds associated with them, including *C. album*, *P. oleracea* and *E. crus-galli* [16, 49, 86]. Maize and rice seem to perform better in controlled conditions, but the interspecific variability and genetic plasticity of weeds could make them more competitive in the field, where many environmental factors interact. One of the possible ways to overcome this and improve the crop performance in field conditions could potentially involve the application of the halotolerant bacteria with plant growth promoting activity. Coexistence of these bacteria with crop species has shown promising results in improving crop resilience to salinity [87].

Our study shows that temperature plays an important role in how salinity stress affects germination and early seedling growth, and highlights that the effect of the temperature-salinity interaction is species-specific, which is expected given the high intraspecific variability. We also found that rising salinity levels had a negative effect on both germination and seedling growth, although in most cases this effect was partially mitigated by higher temperatures. Interestingly, certain species commonly growing in arid regions with high temperatures, such as *Eruca sativa*, *Cyamopsis tetragonoloba* and *Vigna radiata*, exhibit higher tolerance to salinity stress. These species have elevated enzymatic activity and accumulate various antioxidants and osmolytes in leaves and roots [88, 89]. These adaptations may help these plants in overcoming the negative effect of salinity stress by reducing ROS levels.

The most detrimental combination was high salinity and low temperature. The weed species tested had a high degree of salt tolerance either as a reduction in the low germination percentage (*C. album*) or in shoot growth and root elongation (*E. crus-galli, P. oleracea*). This finding suggests that these weed species could be important competitors with crops grown on saline or salt-affected soils, especially at higher temperatures, intensifying their negative effects on crop development. On the other hand, two of the crop species tested, maize and rice, also showed tolerance to high salinity levels at the germination and growth stages. In view of this, one of the ways to suppress or reduce crop-weed competition in saline soils might be to grow salinity-tolerant crop cultivars, given that crops usually tend to germinate at a homogeneous rate, grow fast, and create large canopies that shade the weeds. This can be seen in the case of maize, the germination and growth of which seem to be stimulated by low salinity concentrations.

## Conclusions

Our results suggest that under increasing salinity the competitiveness between weeds and crops could be relevant based on the effects recorded at the seed germination stage and early development. The competitiveness can become even more stringent at high temperatures. Therefore, it is important to control weeds at the early stage of crop development. In view of the increase of salinity and temperature levels due to climate changes and of scarce water resources for irrigation in arid and semi-arid lands, breeding efforts and marker-assisted selection are needed to generate salinity-tolerant cultivars incorporating weed-competitive traits, which is particularly important for crops that are highly salt-sensitive, such as soybean. The salt-tolerant cultivars can be used in weed pre- and post- management control in combination with other strategies. For instance, the use of beneficial microorganisms or the exogenous application of hormones (e.g. salicylic acid) and osmoprotectants to crops could be valuable approaches to increase the crop tolerance to salt stress, thereby making them more competitive against weeds.

More studies should investigate (i) the germination and seedling response of other weed species that could infest crops grown on saline soils, and (ii) the competition between crops and different weed species in the further stages of development. In this regard, soil experiments in controlled saline conditions could confirm our hypothesis of increased weed competitiveness in saline environments. In addition, studies concerning the influence of salinity on soil seedbank might give us further insights into possible development of future weed infestation.

## Methods

### Seed collection and saline solution preparation

Mature seeds of three summer weed species, *Chenopodium album*, *Portulaca oleracea*, and *Echinochloa crus-galli*, common in the fields of soybean, rice and maize of Northern Italy, were collected from September to November 2020 at the Experimental Farm of the University of Padua in Legnaro (north-eastern Italy, 45°12’N, 11°58’E, 6 m above sea level). They were hand-harvested on warm dry days by shaking into paper bags to ensure that only mature seeds were collected. The seeds were then hand cleaned and stored in paper bags at room temperature until the start of trials the following spring. For the crops, three major species were chosen: maize (*Zea mays*) variety DKC5530, soybean (*Glycine max*) variety P21T45, and rice (*Oryza sativa*) variety Vialone Nano.

The saline solutions were prepared by adding pure sodium chloride (NaCl) to distilled water until the desired salinity level was reached, which was measured with an XS Instruments COND 80 electrical conductivity meter (Giorgio Bormac s.r.l, Carpi, Italy) at a sensitivity of 1 µS. Four different saline solutions were prepared: 4, 8, 12 and 16 dS/m, in order to reach these salinity levels 3.20 g/l; 5,50 g/l; 8,50 g/l and 12, 80 g/l of NaCl were added to distilled water. The control consisted of pure distilled water (0 dS/m).

### Germination test

Germination tests for each species, treated with NaCl and untreated, were conducted in three climate chambers with a 12 h light/12 h dark photoperiod, and three different constant temperatures of 12 °C, 15 and 18 °C, simulating early spring conditions of the soil. Four biological replicates (1 replicate = 1 plate) were used for each species at each salinity/temperature combination. Therefore, 60 replicates were prepared for each species (5 salinity levels x 3 temperatures x 4 replicates). Each replicate consisted of 100 seeds in the case of the weeds, 50 in the case of the crops. The seeds were placed in 9 cm-diameter Petri dishes (14 cm-diameter for maize and soybean because of the larger seed size) lined with filter paper and moistened till filter paper was fully imbibed with saline solution (or distilled water for the controls). After placing the seeds on filter paper, the Petri dishes were sealed with parafilm and set inside the climate chambers according to a randomized design. Their position was exchanged every other day. Germination was monitored every 2–3 days, the seeds were considered germinated when a radicle of 1 mm or longer was developed, and was considered completed if all the seeds germinated or if 10 days elapsed without germination, as proposed by [90]. Upon completion, newly germinated seeds were counted and removed. Prior to the test described, preliminary germination tests were conducted for all species and temperatures included in the experiment, during which *E. crus-galli* showed some degree of dormancy (data not shown). *E. crus-galli* is known for having dormancy that can be overcome by seed scarification with sulfuric acid [91]. Therefore, seeds of *E. crus-galli* were immersed in 98% sulfuric acid for 20 min and then thoroughly rinsed. In order to prevent imbibition of the seeds with water, those meant for the trials with saline solutions were rinsed with those solutions after the acid scarification process.

### Preparation of saline nutritive agar base and growth tests

To assess the effects of salinity and temperature on early seedling development, growth tests were conducted by sowing seeds in half-strength MS agar medium [92] without addition of sucrose and hormones, inside plastic containers 8 cm (height) x 9 cm (width) x 10 cm (length). NaCl was added to the MS medium until the required salinity level was reached: 4, 8, 12 or 16 dS/m measured with an XS Instruments COND 80 electrical conductivity meter (Giorgio Bormac s.r.l). The containers were then closed with their original covers and autoclaved for 20 min at 120 °C, then left to cool down. This procedure was followed for both weeds and crops.

In line with the size of the containers and the size of the seeds, 20 crop seeds and 50 weed seeds were sown per container. Salinity and temperature levels were the same as for the germination test, and four replicates were used for each species at each salinity/temperature combination. To prevent contamination of the agar medium, sowing took place inside a sterile environment under a laminar flow hood, and prior to sowing all seeds were sterilized for 30 s with 75% ethanol, followed by a 15-minute treatment with 15% (v/v) sodium hypochlorite (NaClO). The seeds were then washed in distilled water for 5 × 5 min. Once the sowing was completed, the containers were placed inside the climate chambers. In accordance with their different growing speeds, the growth of the crop species was measured after two weeks, the weed species after five weeks. After the established growth period, the plants were carefully removed from the containers, and their stem and root elongation were measured with a digital caliper (TESA Technology, Renens, Switzerland).

### Statistical analysis

For the germination tests, mean germination time (MGT) was calculated using the formula proposed by [93]:

MGT = Σ(nD) / Σn.

where D is the number of days since the start of the test, and n is the number of newly germinated seeds at day D, in accordance also with [94]. The effects of the three factors (species, salinity and temperature) on the germination percentage and on MGT were assessed with a factorial analysis of variance (ANOVA) after a Bartlett homogeneity test. Mean differences were analyzed with a Fisher’s LSD test (α = 0.05). All data analyses were conducted with TIBCO Statistica 14.0.0 software.

### Electronic supplementary material

Below is the link to the electronic supplementary material.

Supplementary Material 1

Supplementary Material 2

Supplementary Material 3

Supplementary Material 4

Supplementary Material 5

Supplementary Material 6

Supplementary Material 7

## Data Availability

The datasets used and/or analysed during the current study are available from the corresponding author on reasonable request.
