# Supplementary material for: Effects of the salinity-temperature interaction on seed germination and early seedling development: a comparative study of crop and weed species
Source: BMC Plant Biol. 2023 Sep 22;23:446. doi: 10.1186/s12870-023-04465-8 (PMC10515249; doi:10.1186/s12870-023-04465-8)
Supplement: Supplementary file 1 — Supplementary Material 1 [file 12870_2023_4465_MOESM1_ESM.docx]

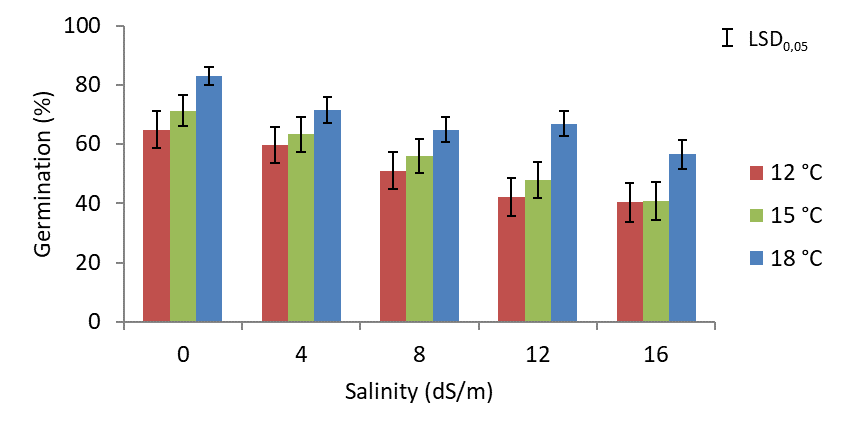


**Figure 1.** Germination percentages of all species tested at different salinity levels and temperatures. Bars indicate the standard errors.
