# Supplementary material for: Effects of the salinity-temperature interaction on seed germination and early seedling development: a comparative study of crop and weed species
Source: BMC Plant Biol. 2023 Sep 22;23:446. doi: 10.1186/s12870-023-04465-8 (PMC10515249; doi:10.1186/s12870-023-04465-8)
Supplement: Supplementary file 2 — Supplementary Material 2 [file 12870_2023_4465_MOESM2_ESM.docx]

**Table 4.** MGT of the three weed species CHEAL (*Chenopodium album*), ECHCG (*Echinochloa crus-galli*), POROL (*Portulaca oleracea*). On different salinity levels and different temperatures.

| Temperature | | 12°C | | 15°C | | 18°C | |
| --- | --- | --- | --- | --- | --- | --- | --- |
| Species | Salinity dS/m | MGT | Err.Std | MGT | Err.Std | MGT | Err.Std |
| CHEAL | 0 | 11 | 0,43 | 10 | 0,11 | 8 | 0,24 |
| CHEAL | 4 | 13 | 0,34 | 11 | 0,25 | 9 | 0,25 |
| CHEAL | 8 | 11 | 0,52 | 12 | 0,32 | 10 | 0,43 |
| CHEAL | 12 | 13 | 0,33 | 11 | 0,83 | 11 | 0,20 |
| CHEAL | 16 | 14 | 0,22 | 12 | 0,69 | 11 | 0,16 |
| ECHCG | 0 | 15 | 1,56 | 11 | 0,17 | 8 | 0,19 |
| ECHCG | 4 | 17 | 0,79 | 12 | 0,53 | 8 | 0,13 |
| ECHCG | 8 | 18 | 1,12 | 15 | 1,18 | 8 | 0,14 |
| ECHCG | 12 | 19 | 2,95 | 12 | 1,08 | 10 | 0,27 |
| ECHCG | 16 | 21 | 1,17 | 17 | 1,46 | 9 | 0,06 |
| POROL | 0 | 7 | 0,09 | 6 | 0,09 | 4 | 0,11 |
| POROL | 4 | 6 | 0,04 | 6 | 0,16 | 4 | 0,07 |
| POROL | 8 | 8 | 0,01 | 7 | 0,06 | 5 | 0,21 |
| POROL | 12 | 11 | 0,09 | 7 | 0,06 | 6 | 0,15 |
| POROL | 16 | 9 | 0,34 | 8 | 0,26 | 7 | 0,15 |
