# Supplementary material for: Effects of the salinity-temperature interaction on seed germination and early seedling development: a comparative study of crop and weed species
Source: BMC Plant Biol. 2023 Sep 22;23:446. doi: 10.1186/s12870-023-04465-8 (PMC10515249; doi:10.1186/s12870-023-04465-8)
Supplement: Supplementary file 3 — Supplementary Material 3 [file 12870_2023_4465_MOESM3_ESM.docx]

**Table 1.** Germination percentage of the three crop species MAIZE (*Zea mays*), RICE (*Oryza sativa*), SOY (*Glycine max*) at different salinity levels and different temperatures.

| Temperature | | 12°C | | 15°C | | 18°C | |
| --- | --- | --- | --- | --- | --- | --- | --- |
| Species | Salinity dS/m | Germination % | Err.Std | Germination % | Err.Std | Germination % | Err.Std |
| MAIZE | 0 | 97.5 | 0.96 | 99 | 1.00 | 94 | 1.63 |
| MAIZE | 4 | 98 | 0.82 | 98.5 | 0.96 | 99 | 1.00 |
| MAIZE | 8 | 97 | 1.29 | 91.5 | 2.75 | 95 | 0.58 |
| MAIZE | 12 | 90 | 3.46 | 90.5 | 2.22 | 93.5 | 2.22 |
| MAIZE | 16 | 92.5 | 2.99 | 89.5 | 1.26 | 88.5 | 4.11 |
| RICE | 0 | 85 | 3.11 | 85.5 | 1.50 | 90 | 1.41 |
| RICE | 4 | 77 | 1.29 | 91.5 | 0.50 | 94 | 1.63 |
| RICE | 8 | 82.5 | 3.59 | 86.5 | 1.26 | 86.5 | 2.22 |
| RICE | 12 | 76 | 3.92 | 79 | 3.87 | 88 | 0.82 |
| RICE | 16 | 71.5 | 3.20 | 75 | 2.38 | 87 | 1.29 |
| SOY | 0 | 92 | 1.63 | 94 | 2.58 | 91.5 | 2.06 |
| SOY | 4 | 71 | 7.94 | 65.5 | 6.34 | 46.5 | 7.93 |
| SOY | 8 | 50.5 | 5.56 | 61 | 4.65 | 58.5 | 6.34 |
| SOY | 12 | 26.5 | 4.92 | 50 | 0.82 | 41.5 | 5.50 |
| SOY | 16 | 27.5 | 2.50 | 20 | 4.40 | 40 | 4.55 |
