# Supplementary material for: Effects of the salinity-temperature interaction on seed germination and early seedling development: a comparative study of crop and weed species
Source: BMC Plant Biol. 2023 Sep 22;23:446. doi: 10.1186/s12870-023-04465-8 (PMC10515249; doi:10.1186/s12870-023-04465-8)
Supplement: Supplementary file 4 — Supplementary Material 4 [file 12870_2023_4465_MOESM4_ESM.docx]

**Table 2.** Germination percentage of the three weed species CHEAL (*Chenopodium album*), ECHCG (*Echinochloa crus-galli*), POROL (*Portulaca oleracea*) at different salinity levels and different temperatures.

| Temperature | | 12°C | | 15°C | | 18°C | |
| --- | --- | --- | --- | --- | --- | --- | --- |
| Species | Salinity dS/m | Germination % | Err.Std | Germination % | Err.Std | Germination % | Err.Std |
| CHEAL | 0 | 32 | 3.56 | 33.5 | 3.59 | 58 | 3.37 |
| CHEAL | 4 | 21 | 4.65 | 32.5 | 2.87 | 59.5 | 5.38 |
| CHEAL | 8 | 22 | 1.83 | 38.5 | 4.50 | 44 | 4.97 |
| CHEAL | 12 | 23 | 2.38 | 27.5 | 3.10 | 68 | 6.78 |
| CHEAL | 16 | 27 | 5.20 | 24.5 | 3.10 | 44.5 | 4.79 |
| ECHCG | 0 | 58.5 | 5.91 | 71.5 | 7.04 | 72 | 3.74 |
| ECHCG | 4 | 65 | 2.65 | 66 | 2.94 | 72 | 3.56 |
| ECHCG | 8 | 37.5 | 5.74 | 44.5 | 3.10 | 48.5 | 3.30 |
| ECHCG | 12 | 24 | 6.38 | 29.5 | 2.63 | 56.5 | 3.30 |
| ECHCG | 16 | 19.5 | 4.11 | 30 | 5.60 | 36.5 | 0.96 |
| POROL | 0 | 24.5 | 5.19 | 44.5 | 3.59 | 92 | 5.23 |
| POROL | 4 | 25.5 | 7.89 | 25.5 | 2.50 | 58 | 4.97 |
| POROL | 8 | 17 | 5.07 | 14 | 2.31 | 56.5 | 3.59 |
| POROL | 12 | 13 | 4.43 | 11.5 | 3.86 | 53.5 | 5.19 |
| POROL | 16 | 4.5 | 1.71 | 6.5 | 2.22 | 43 | 7.59 |
