# Supplementary material for: Effects of the salinity-temperature interaction on seed germination and early seedling development: a comparative study of crop and weed species
Source: BMC Plant Biol. 2023 Sep 22;23:446. doi: 10.1186/s12870-023-04465-8 (PMC10515249; doi:10.1186/s12870-023-04465-8)
Supplement: Supplementary file 5 — Supplementary Material 5 [file 12870_2023_4465_MOESM5_ESM.docx]

**Table 3.** MGT of the three crop species MAIZE (*Zea mays*), RICE (*Oryze sativa*), SOY (*Glicine max*). On different salinity levels and different temperatures.

| Temperature | | 12°C | | 15°C | | 18°C | |
| --- | --- | --- | --- | --- | --- | --- | --- |
| Species | Salinity dS/m | MGT | Err.Std | MGT | Err.Std | MGT | Err.Std |
| MAIZE | 0 | 10 | 0.55 | 8 | 0.08 | 6 | 0.30 |
| MAIZE | 4 | 9 | 0.36 | 8 | 0.27 | 6 | 0.37 |
| MAIZE | 8 | 12 | 0.73 | 8 | 0.25 | 6 | 0.35 |
| MAIZE | 12 | 11 | 0.95 | 9 | 0.42 | 6 | 0.33 |
| MAIZE | 16 | 12 | 4.12 | 9 | 3.27 | 8 | 0.26 |
| RICE | 0 | 9 | 0.60 | 8 | 0.14 | 6 | 0.14 |
| RICE | 4 | 10 | 0.39 | 8 | 0.29 | 6 | 0.10 |
| RICE | 8 | 11 | 0.49 | 8 | 0.08 | 6 | 0.06 |
| RICE | 12 | 11 | 0.44 | 8 | 0.35 | 7 | 0.13 |
| RICE | 16 | 13 | 0.48 | 9 | 0.32 | 7 | 0.08 |
| SOY | 0 | 9 | 0.22 | 9 | 0.44 | 6 | 0.21 |
| SOY | 4 | 11 | 0.79 | 9 | 0.52 | 9 | 0.62 |
| SOY | 8 | 12 | 0.63 | 10 | 0.24 | 9 | 0.75 |
| SOY | 12 | 15 | 0.56 | 13 | 0.52 | 9 | 0.18 |
| SOY | 16 | 16 | 0.12 | 13 | 0.84 | 6 | 0.22 |
