# Supplementary material for: Effects of the salinity-temperature interaction on seed germination and early seedling development: a comparative study of crop and weed species
Source: BMC Plant Biol. 2023 Sep 22;23:446. doi: 10.1186/s12870-023-04465-8 (PMC10515249; doi:10.1186/s12870-023-04465-8)
Supplement: Supplementary file 6 — Supplementary Material 6 [file 12870_2023_4465_MOESM6_ESM.docx]

**Table 5.** Stem and root length of control (0 dS/m) of the six species used in the trial

| **Crops** | **Temperature (°C)** | **Stem lenght (mm)** | **± err.st** | **Root length (mm)** | **± err.st** |
| --- | --- | --- | --- | --- | --- |
| Maize | 12 | 13,5 | 0,40 | 16,6 | 0,71 |
| Maize | 15 | 24,6 | 1,16 | 23,8 | 0,76 |
| Maize | 18 | 38,9 | 1,71 | 24,4 | 0,77 |
| Rice | 12 | 4,9 | 0,29 | 2,2 | 0,32 |
| Rice | 15 | 8,2 | 0,25 | 9,4 | 0,81 |
| Rice | 18 | 24,5 | 1,88 | 24,1 | 1,19 |
| Soybean | 12 | 0,0 | 0,00 | 52,8 | 2,20 |
| Soybean | 15 | 46,6 | 3,65 | 29,1 | 1,07 |
| Soybean | 18 | 79,6 | 4,51 | 33,5 | 1,99 |
| **Weed species** | **Temperature (°C)** | **Stem lenght (mm)** | **± err.st** | **Root length (mm)** | **± err.st** |
| Chenopodium album | 12 | 36,4 | 1,50 | 8,3 | 0,43 |
| Chenopodium album | 15 | 40,8 | 1,08 | 11,1 | 0,39 |
| Chenopodium album | 18 | 39,6 | 0,74 | 14,3 | 0,45 |
| Echinochloa crus-galli | 12 | 30,5 | 1,02 | 14,9 | 0,53 |
| Echinochloa crus-galli | 15 | 39,1 | 1,60 | 12,9 | 0,64 |
| Echinochloa crus-galli | 18 | 51,4 | 2,39 | 16,2 | 0,78 |
| Portulaca oleracea | 12 | 5,4 | 0,17 | 2,8 | 0,11 |
| Portulaca oleracea | 15 | 9,2 | 0,30 | 3,9 | 0,09 |
| Portulaca oleracea | 18 | 9,2 | 0,18 | 5,9 | 0,16 |
