# Supplementary material for: Effects of the salinity-temperature interaction on seed germination and early seedling development: a comparative study of crop and weed species
Source: BMC Plant Biol. 2023 Sep 22;23:446. doi: 10.1186/s12870-023-04465-8 (PMC10515249; doi:10.1186/s12870-023-04465-8)
Supplement: Supplementary file 7 — Supplementary Material 7 [file 12870_2023_4465_MOESM7_ESM.docx]

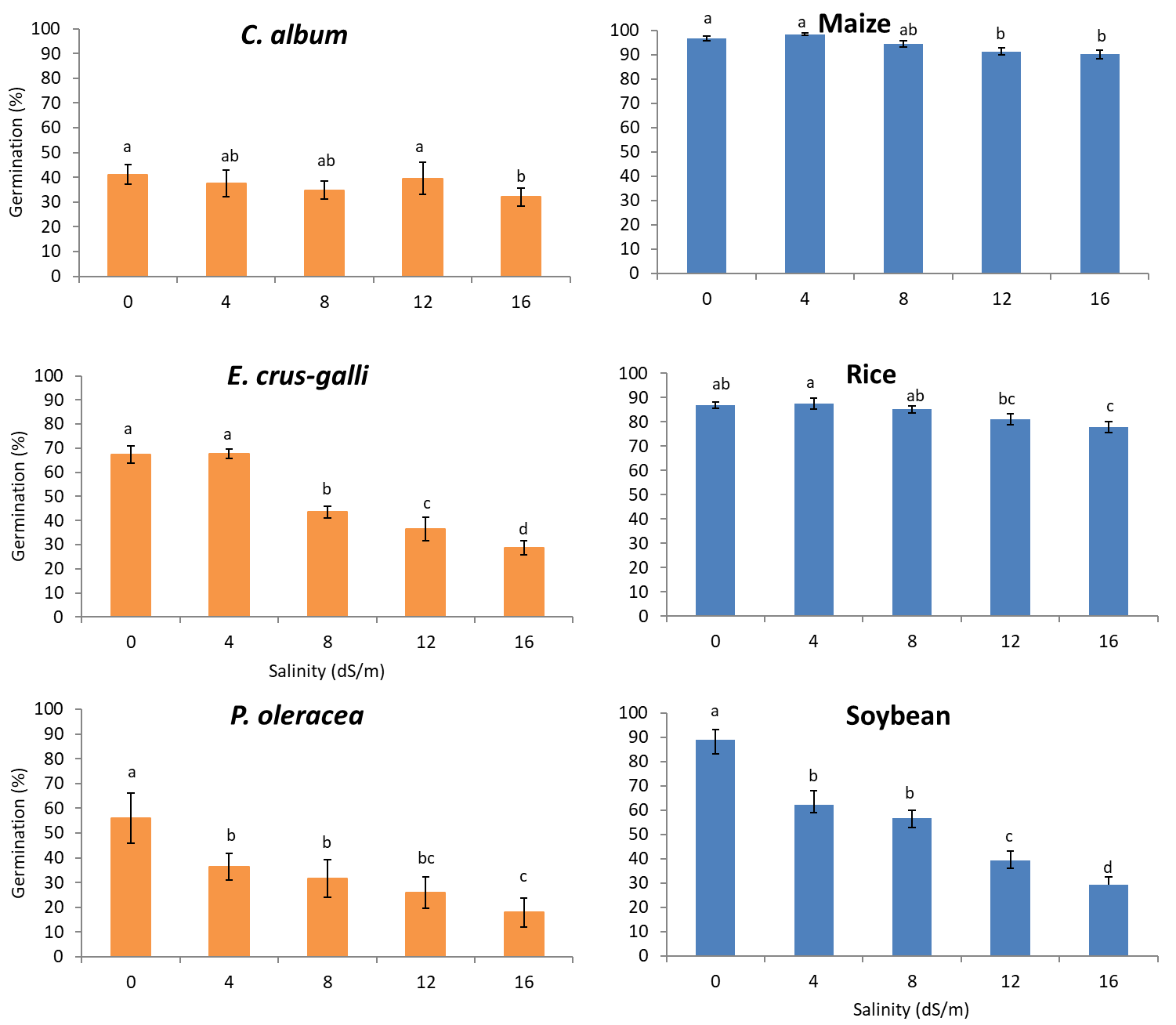


**Figure 2.** Germination percentages of the three weed species *Chenopodium album*, *Echinochloa crus-galli*, and *Portulaca oleracea*, and the three crop species Maize (*Zea mays*), Rice (*Oryze sativa*), and Soybean (*Glycine max*) at different salinity levels. Letters indicate significant differences (α = 0.05).
